# The influence of structure on the response properties of biologically plausible neural network models

**DOI:** 10.1186/1471-2202-12-S1-P30

**Published:** 2011-07-18

**Authors:** Christian Tomm, Michael Avermann, Tim Vogels, Wulfram Gerstner, Carl Petersen

**Affiliations:** 1 School of Computer and Communication Sciences and Brain-Mind Institute, Ecole Polytechnique Federale de Lausanne, 1015 Lausanne EPFL, Switzerland; 2 Friedrich Miescher Institute for Biomedical Research, Maulbeerstrasse 66, 4058 Basel, Switzerland; 3 Brain-Mind Institute, Ecole Polytechnique Federale de Lausanne, 1015 Lausanne EPFL, Switzerland

## 

Random sparse network architectures are intriguingly easy to construct, and, due to the lack of relevant structural studies, they have been difficult to disprove. Over the last decade, they have become the workhorse of computational studies of neuronal network dynamics. With the arrival of optical methods like channelrhodopsin and glutamate uncaging we can finally compare the predictions stemming from these architectures with responses measured in real cortical networks.

Our model network is simulating L2/3 of a mouse barrel cortex column with three distinct neuronal populations: Excitatory pyramidal cells, fast-spiking inhibitory cells and non-fast-spiking inhibitory cells, modeled with biological plausible, cell-type specific adaptive exponential integrate and fire (AdEx) models [1]. For the connections between the three populations we used the probability of each connection and the distribution of synaptic weights, observed in biological measurements [2].

In parallel experiments and simulations, in which we stimulate groups of neurons and record the responses of the surrounding network, we can show that uniform random network models constructed from the statistics of pairwise recordings cannot capture the experimentally measured network responses.

To improve the similarity between model and experiments, we investigate different ways to change the network architecture, for example by introducing correlations in the distribution of synaptic weights [3] or by modifying the in- and out-degree distributions of the network neurons. These changes are applied in a way that will still preserve the pairwise statistics in regards to the strengths and probabilities measured in biological experiments. In order to avoid an explosion of the number of usable parameters we are focusing on changes of the three most relevant types of connections, excitatory to excitatory, fast-spiking and non-fast-spiking, leading to a 12 dimensional parameter space. In order to visualize the high dimensional parameter space we rely on clutter based dimension reordering (CBDR) [4], a graphical technique used to build an intuitive representation of high dimensional data. This allows us to find not only the best network architecture in our parameter space but also to give an intuitive understanding of the impact of each parameter and of the interactions between parameters. The parameters with the highest impact on the response similarity between model and experiment are the in-degree and the input weight correlations in the connection from excitatory to fast-spiking neurons. Our work should contribute to a new, standard model architecture that will replace random networks as a tool to analyze biological network behavior.
